# Hyperglycemic Hyperosmolar State As the Initial Presentation of Type 1 Diabetes Mellitus in an Adolescent: A Case Report

**DOI:** 10.7759/cureus.116085

**Published:** 2026-09-11

**Authors:** Paula García-Sánchez, Amalia García Durán, Patricia Bote Gascón, Nerea Itza Martín, María de Ceano-Vivas La Calle

**Affiliations:** 1 Pediatric Emergency Department, Hospital Universitario La Paz, Madrid, ESP; 2 Pediatric Endocrinology Department, Hospital Universitario La Paz, Madrid, ESP

**Keywords:** diabetes mellitus, diabetic ketoacidosis, hyperglycemia, hyperglycemic hyperosmolar state, hyperosmolality, insulin

## Abstract

Hyperglycemic hyperosmolar state (HHS) is a rare but life-threatening hyperglycemic emergency in children and adolescents. It is characterized by severe hyperglycemia, hyperosmolality, and dehydration, with absent or limited ketoacidosis. Although more commonly associated with type 2 diabetes mellitus, HHS may also occur as the initial presentation of type 1 diabetes mellitus in pediatric patients.

We report the case of a previously healthy 13-year-old boy presenting with a three-week history of polydipsia and nocturnal polyuria. Initial evaluation revealed profound hyperglycemia (>1,000 mg/dL), hyperosmolality, clinical dehydration, and only mild acidosis. During treatment, the patient experienced marked fluctuations in blood glucose, requiring repeated adjustments of insulin and dextrose infusions, electrolyte replacement, and pediatric intensive care monitoring.

This case highlights the diagnostic challenges of pediatric HHS and emphasizes the importance of early recognition, prioritizing fluid resuscitation, cautious insulin administration, and close biochemical monitoring to prevent complications.

## Introduction

Hyperglycemic hyperosmolar state (HHS) is a rare but life-threatening acute metabolic complication of diabetes mellitus in children and adolescents. It is characterized by profound hyperglycemia, severe dehydration, hyperosmolality, and absent or only mild ketoacidosis, distinguishing it from the more common presentation of diabetic ketoacidosis (DKA) [1-3]. Although HHS has classically been associated with type 2 diabetes mellitus, an increasing number of pediatric cases have been reported as the initial manifestation of type 1 diabetes [2-5].

Pediatric HHS differs from DKA not only in its biochemical profile but also in its clinical course and therapeutic considerations [6,7]. Compared with DKA, HHS is associated with higher morbidity and mortality, emphasizing the importance of early recognition, careful fluid management, and close biochemical and neurological monitoring [3,8,9].

We report the case of a previously healthy adolescent presenting with HHS as the first manifestation of type 1 diabetes mellitus. This case highlights the diagnostic challenges posed by overlapping features of HHS and DKA, the complexity of metabolic management, and the importance of careful monitoring during treatment.

## Case presentation

A previously healthy 13-year-old boy presented to the Pediatric Emergency Department (PED) of a tertiary care hospital in Madrid, Spain, with a three-week history of progressive polydipsia and nocturnal polyuria. He also reported intermittent abdominal pain without vomiting or diarrhea and mild bilateral calf pain. No significant recent weight loss was reported by the patient or his family. At symptom onset, he had experienced a dental abscess that had been managed by his dentist. His medical history was significant only for a surgically treated radius and ulna fracture two months earlier. He had no previous endocrine disorders, chronic illnesses, or relevant family history.

At triage, the patient was categorized as level 2 (medical attention in less than 15 minutes) and was found to be hemodynamically stable but clinically dehydrated. Vital signs showed a heart rate of 84 beats/min, blood pressure of 109/68 mmHg, and oxygen saturation of 98% on room air. Anthropometric measurements showed a weight of 46.1 kg (31st percentile), a height of 158.3 cm (47th percentile), and a body mass index (BMI) of 18.4 kg/m^2^ (30th percentile). On physical examination, he was pale and had dry oral mucous membranes. Neurologically, he was alert and oriented, with a Glasgow Coma Scale score of 15/15. No focal neurological deficits were identified. Point-of-care capillary glucose exceeded the upper detection limit of the glucometer ("High").

Initial laboratory investigations demonstrated severe hyperglycemia (plasma glucose, 1,029 mg/dL), ketosis (blood ketones, 5.6 mmol/L), venous pH of 7.28, bicarbonate of 20.6 mmol/L, serum sodium of 123 mmol/L (corrected sodium, 145 mmol/L), calculated serum osmolality of 318 mOsm/L, and serum creatinine of 0.7 mg/dL. Complete blood count findings were unremarkable. Table 1 summarizes the main laboratory findings on the patient’s arrival at the PED.

**Table 1:** Initial laboratory findings at presentation

Parameters	Patient values	Reference range
Hemoglobin (g/dL)	14.1	12.5-17.2
White blood cell count (×10^9^/L)	5.9	3.8-10.3
Platelet count (×10^9^/L)	333	174-409
Sodium (mmol/L)	123	136-145
Corrected sodium (mmol/L)	145	136-145
Potassium (mmol/L)	5.1	3.5-5.1
Chloride (mmol/L)	86	99-109
Magnesium (mg/dL)	2.2	1.6-2.6
Calcium (mg/dlL)	10.3	9.1-10.2
Phosphate (mg/dL)	5.3	4-5.8
Serum osmolality (mOsm/L)	318	275-295
Blood glucose (mg/dL)	1,029	74-106
Blood ketones (mmol/L)	5.6	<1
Creatinine (mg/dL)	0.7	0.5-1
C-reactive protein (mg/L)	<3	<5
pH	7.28	7.33-7.43
Bicarbonate (mmol/L)	20.6	23-27

Fluid resuscitation was initiated with 0.9% saline at 10 mL/kg over one hour, resulting in a decrease in blood glucose to 810 mg/dL. Continuous intravenous insulin infusion was subsequently started, with serial monitoring of blood glucose, venous blood gases, electrolytes, and ketone concentrations. During the initial hours of treatment, blood glucose decreased rapidly to 461 mg/dL, requiring frequent adjustments of the insulin infusion. Figure 1 shows the initial trend in blood glucose measurements during the patient’s stay in the PED.

**Figure 1:**
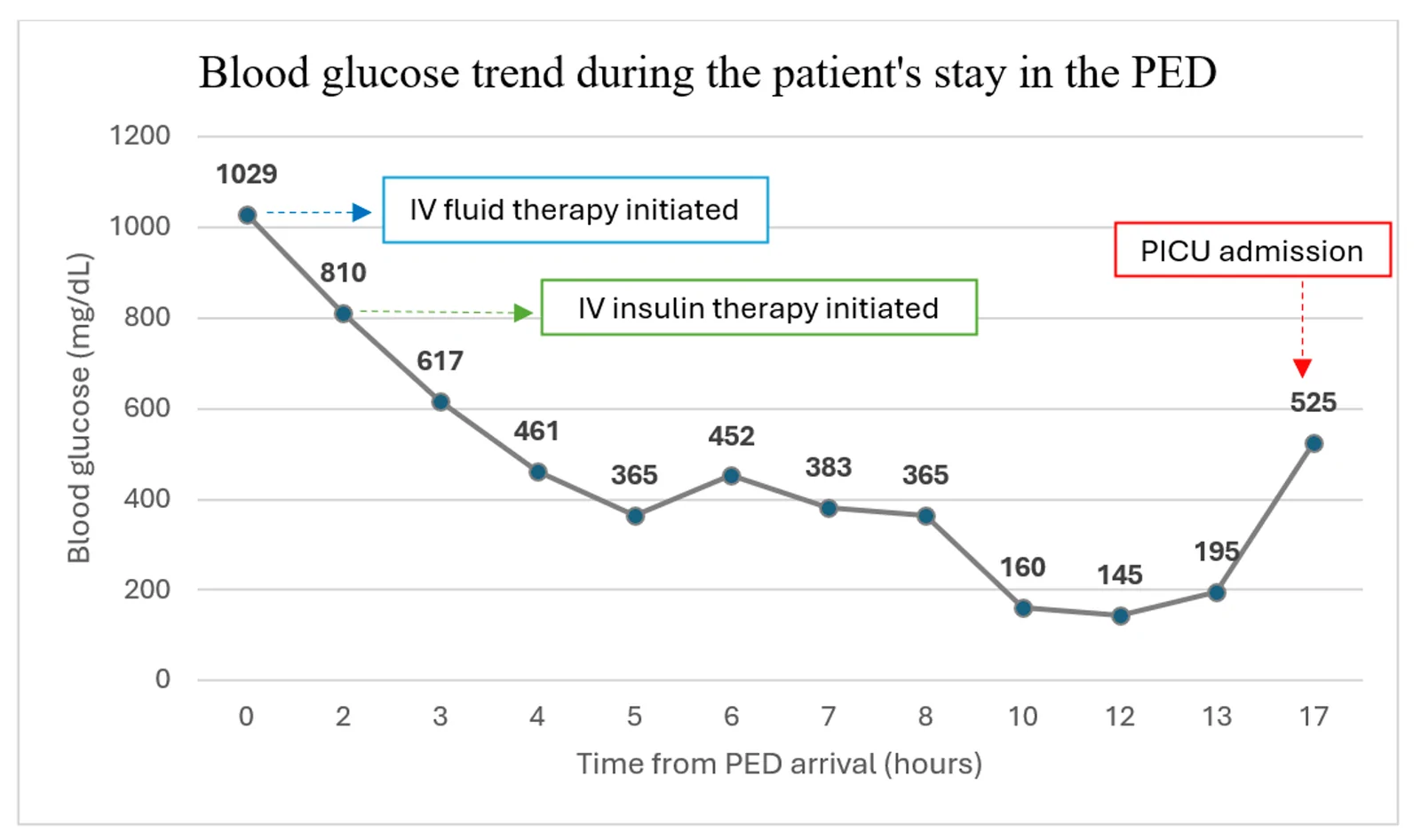
Blood glucose trend during the patient's stay in the PED PED: Pediatric Emergency Department; IV: intravenous; PICU: Pediatric Intensive Care Unit

Coinciding with a decrease in blood glucose to 365 mg/dL, the patient developed painful muscle cramps involving both calves and the fingers and toes, accompanied by intermittent abdominal pain. Frequent adjustments of the intravenous insulin infusion and dextrose supplementation were required because of marked glycemic fluctuations. The patient also required intravenous potassium and magnesium replacement during treatment in the PED.

Despite these interventions, metabolic control remained difficult to achieve, prompting admission to the Pediatric Intensive Care Unit (PICU) for close monitoring and ongoing management. He remained in the PICU for 24 hours, during which metabolic control progressively stabilized with continuous intravenous insulin, fluid therapy, and electrolyte replacement. He was then transitioned to a basal-bolus subcutaneous insulin regimen and transferred to the pediatric ward.

The patient remained hospitalized for an additional four days to complete structured diabetes education and insulin administration training before discharge.

## Discussion

This case illustrates the diagnostic and therapeutic challenges of HHS in the pediatric population. Although DKA accounts for the vast majority of hyperglycemic emergencies in children, our patient presented with extreme hyperglycemia (>1,000 mg/dL), hyperosmolality, moderate dehydration, and only mild acidosis despite significant ketonemia, a constellation of findings more consistent with HHS than with classic DKA [3,6-8]. As in previously reported pediatric cases, the relatively preserved acid-base status may contribute to delayed recognition because patients often appear clinically less ill than expected for the degree of metabolic derangement. Table 2 compares pediatric DKA and HHS.

**Table 2:** Comparison of pediatric DKA and HHS “Current case” refers to the patient described in this case report. Although the calculated serum osmolality was elevated at 318 mOsm/kg, slightly below the conventional threshold of 320 mOsm/kg, the overall clinical and biochemical presentation - including extreme hyperglycemia, significant dehydration, and relatively preserved acid-base status despite marked ketonemia - was considered more consistent with an HHS phenotype than with classic DKA. DKA: diabetic ketoacidosis; HHS: hyperglycemic hyperosmolar state

	DKA	HHS	Current case
Blood glucose (mg/dL)	>250	>600	1,029
Venous pH	<7.3	>7.25	7.28
Bicarbonate (mmol/L)	<15	>15	20.6
Serum osmolality (mOsm/L)	Variable	>320	318
Ketosis (mmol/L)	Marked	Mild or absent	5.6
Dehydration	Moderate	Severe	Present
Initial treatment priority	Fluids + insulin	Initial fluid resuscitation (followed by cautious insulin therapy)	-
Mortality	Lower	Higher	-

HHS remains an uncommon presentation of diabetes in childhood and has traditionally been associated with type 2 diabetes mellitus [2,3]. However, increasing numbers of reports describe HHS as the initial manifestation of type 1 diabetes, highlighting the importance of considering this diagnosis in any child presenting with profound hyperglycemia and marked dehydration [2,3,5]. Watanabe et al. described a 14-year-old boy with newly diagnosed type 1 diabetes mellitus who presented with a glucose concentration of 1,010 mg/dL and an osmolality of 313 mOsm/kg, without significant acidosis and without ketosis [5]. Similarly, Chun et al. reported an adolescent with newly diagnosed type 1 diabetes mellitus who presented with a glucose concentration of 1,456 mg/dL, marked hyperosmolality, and mild acidosis [1]. Our patient showed a similarly profound degree of hyperglycemia, comparable to the values reported in these previously described pediatric cases, but in contrast to these reports, our patient presented with significant ketonemia (5.6 mmol/L) and mild acidosis (pH 7.28), which may have further complicated the diagnosis. This overlap makes the diagnosis challenging.

In our patient, the preceding dental infection may have acted as a precipitating stressor, triggering the metabolic decompensation. Infection is considered the most common precipitating factor for HHS, accounting for approximately 50-60% of reported cases in some series [8]. However, other potential triggers should also be considered, including some medications, cardiovascular events, and other conditions associated with increased physiological stress [8].

In HHS, relative insulin availability is generally sufficient to limit lipolysis and ketogenesis, while remaining insufficient to prevent severe hyperglycemia [2,3]. Progressive osmotic diuresis results in substantial free-water losses, leading to marked dehydration and hyperosmolality. Consequently, the initial therapeutic priority is vigorous fluid resuscitation with close monitoring to restore circulating volume and improve tissue perfusion before initiating insulin therapy, which should be introduced cautiously to avoid an excessive decline in plasma glucose and serum osmolality. Initial resuscitation is performed with isotonic fluids, typically administered as 20 mL/kg boluses and repeated as needed until adequate circulation and tissue perfusion are restored. After hemodynamic stabilization, patients with persistent hypernatremia and hyperosmolality may be transitioned to hypotonic fluids, ensuring a gradual correction of serum sodium and osmolality to minimize the risk of cerebral edema [1,3,6]. Given the complexity of fluid and metabolic management, and the higher reported mortality compared with DKA, treatment should ideally be carried out in a PICU with continuous clinical and biochemical monitoring [6].

Our patient demonstrated a rapid reduction in blood glucose shortly after initiation of intravenous insulin despite careful fluid resuscitation. This was accompanied by painful muscle cramps and marked glycemic variability, requiring repeated adjustments of insulin and dextrose infusions and ultimately admission to the PICU. Although no neurological complications occurred, this clinical course underscores the need for frequent biochemical monitoring and individualized treatment, with careful fluid management as the therapeutic priority.

Pediatric HHS continues to be associated with significantly higher morbidity and mortality than DKA, largely because of delayed diagnosis, profound dehydration, electrolyte disturbances, and the risk of cerebral edema during treatment [3,7,8].

This case highlights the importance of recognizing HHS as a possible initial presentation of diabetes mellitus in children and reinforces the need for meticulous monitoring during the acute phase of treatment. Although this is a single case with limited generalizability, its clinical relevance lies in the rarity and potentially life-threatening nature of pediatric HHS. Reporting such cases can help raise awareness among healthcare professionals and facilitate earlier recognition and appropriate management of this uncommon condition.

## Conclusions

This case contributes to addressing a gap in the literature by providing further evidence of the rare presentation of HHS in an adolescent with newly diagnosed type 1 diabetes mellitus, emphasizing the diagnostic and therapeutic challenges that may arise in this setting. Despite extreme hyperglycemia and severe dehydration, the patient presented with only mild acidosis, highlighting the potential for delayed recognition of this uncommon pediatric emergency. Early diagnosis, careful fluid resuscitation, cautious insulin administration, and meticulous monitoring are essential to reduce the risk of serious complications and improve clinical outcomes.
